# Supplementary material for: Cardiac Synchrony During Collaborative Drawing: A Longitudinal Comparison of Same Generation and Intergenerational Dyads
Source: Ann N Y Acad Sci. 2026 Apr 7;1558(1):e70272. doi: 10.1111/nyas.70272 (PMC13056352; doi:10.1111/nyas.70272)
Supplement: Supplementary file 1 — Supplementary Materials: nyas70272‐sup‐0001‐SuppMat.pdf [file NYAS-1558-0-s001.pdf]

Supplementary Materials: Moffat, Naudszus and Cross (2025). ***Cardiac synchrony during collaborative drawing: A longitudinal comparison of same generation and intergenerational dyads.***

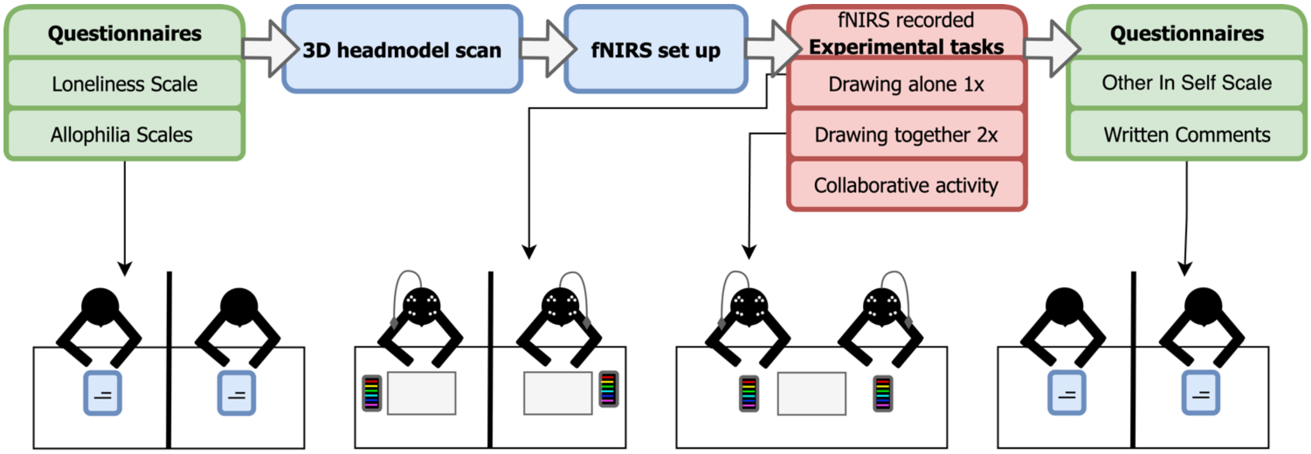

Figure 1. Flow chart showing the protocol employed at each session.

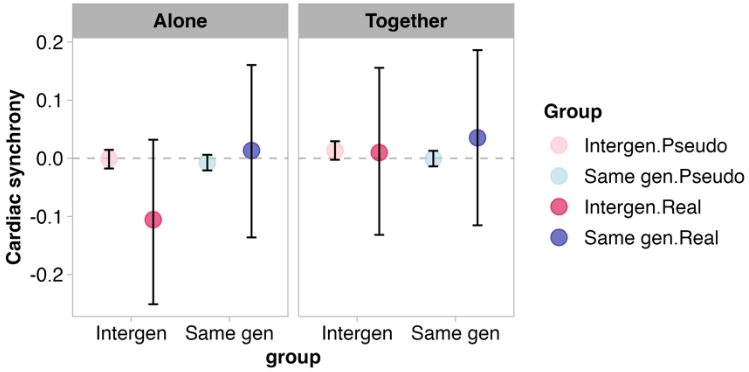

Figure 2. All sessions combined, parameter estimates per group and condition. Error bars show 95 % HPD.

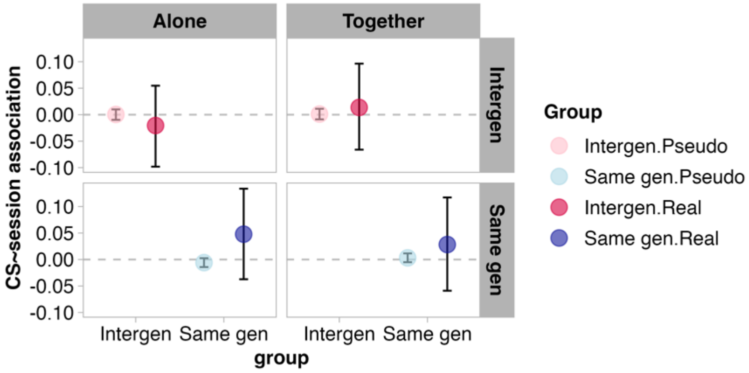

Figure 3. Change in cardiac synchrony (CS) across sessions, parameter estimates per group and condition. Error bars show 95 % HPD.

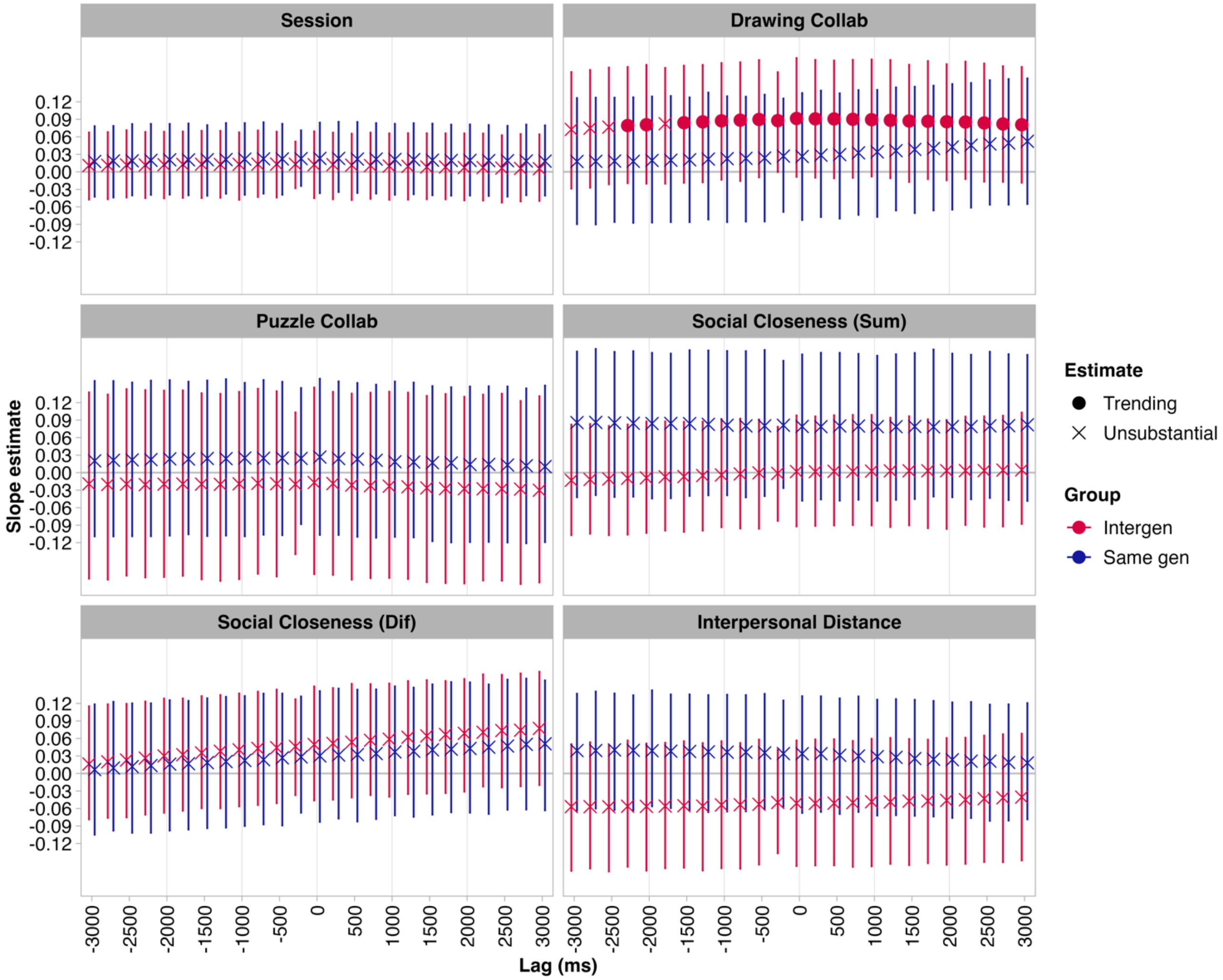

Figure 4. Relationship between cardiac synchrony and measures. Error bars show 95 % HPD.

Table 1. Breakdown of reasons for excluding dyads data. Recordings/dyads only included once in list. E.g., if a recording/dyad is counted among reasons unrelated to signal quality, they are not re-counted among reasons related to signal quality. In total 91 recordings of 732 recordings were excluded, impacting 85 of 366 dyadic sessions.

**Reason**	**N individual recordings excluded**	**N dyadic sessions impacted**
Unrelated to signal quality		
Recordings lost in transition between recording laptops	2	2
Triggers missing or unreliable	7	4
Participant unable to complete session (could not attend final session/consumed alcohol)	5	3
Multi-part recording (bluetooth or battery dropout)	6	4
Related to signal quality		
No short channels of adequate quality	71	69
No long channels of adequate quality (pilot montage used)	0	0
**Total**	**91**	**85**

Table 2. Standardised estimates of relationship between behavioural and self-report measure per group.

	**Drawing Collaboration**	**Puzzle Collaboration**	**Social Closeness (Sum)**	**Social Closeness (Dif)**
**Drawing Collaboration (Intergen)**	-			
**Drawing Collaboration (Samegen)**	-			
**Puzzle Collaboration (Intergen)**	0.19 [0.06, 0.32]	-		
**Puzzle Collaboration (Samegen)**	-0.07 [-0.18, 0.04]	-		
**Social Closeness Sum (Intergen)**	0.19 [0.10, 0.28]	-0.05 [-0.13, 0.02]	-	
**Social Closeness Sum (Samegen)**	0.35 [0.23, 0.46]	-0.23 [-0.33, 0.13]	-	
**Social Closeness Dif (Intergen)**	-0.03 [-0.13, 0.08]	0.06 [-0.02, 0.14]	0.19 [0.11, 0.27]	-
**Social Closeness Dif (Samegen)**	-0.00 [-0.10, 0.11]	-0.15 [-0.23, -0.06]	0.11 [0.02, 0.19]	-
**Proximity (Intergen)**	-0.11 [-0.22, -0.01]	-0.02 [-0.11, 0.05]	0.10 [0.01, 0.19]	-0.09 [-0.19, 0.01]
**Proximity (Samegen)**	0.10 [0.00, 0.20]	-0.09 [-0.17, -0.00]	-0.13 [-0.24, -0.01]	0.12 [0.02, 0.22]
